# Differential effects of PDCD4 depletion on protein synthesis in myoblast and myotubes

**DOI:** 10.1186/1471-2121-15-2

**Published:** 2014-01-09

**Authors:** Dhanshri Kakade, Nushaba Islam, Naomi Maeda, Olasunkanmi A J Adegoke

**Affiliations:** 1 School of Kinesiology & Health Science and Muscle Health Research Centre, York University, 4700 Keele Street, Toronto, Ontario M3J 1P3, Canada

**Keywords:** PDCD4, mRNA translation, S6K1, Protein synthesis, Skeletal muscle

## Abstract

**Background:**

Reduced muscle mass is a hallmark of metabolic diseases like diabetes and cancer. The mammalian (mechanistic) target of rapamycin complex 1/S6 kinase 1 (mTORC1/S6K1) pathway is critical to the regulation of muscle protein synthesis and mass but its mechanism of action is not completely understood.

**Results:**

Using L6 myotubes, we characterized the regulation of programmed cell death 4 (PDCD4), a recently described substrate of S6K1. The abundance, but not Ser67 phosphorylation, of PDCD4 was sensitive to amino acid and serum deprivation: values in starved cells were 4.5X of control (P < 0.001). Refeeding had opposite effects. Growth factors, compared to amino acids, appeared more critical in regulating PDCD4 abundance. Furthermore, inhibition of mTORC1 or the proteasome prevented the refeeding-associated decrease in PDCD4 abundance. Amino acid and serum deprivation significantly increased PDCD4 binding to eIF4A (P < 0.05); this was reversed during refeeding. PDCD4 depletion by RNA interference had no significant effect on phenylalanine incorporation into myotube mixed proteins in control cells but further suppressed (30%) this measure in nutrient-deprived cells (P < 0.0005). This was not observed in myoblasts. In starved myotubes, PDCD4 depletion further reduced the association of eIF4G with eIF4E.

**Conclusion:**

Our data suggest that in myotubes, PDCD4 abundance is sensitive to nutritional manipulation in an mTORC1 and proteasome depended manner. Furthermore, the role of PDCD4 in regulating protein synthesis appears dependent on the developmental state of the cell.

## Background

The mammalian (mechanistic) target of rapamycin complex 1/ribosomal protein S6 kinase 1 (mTORC1/S6K1) signalling is a critical regulator of skeletal muscle mass and metabolism, and mechanisms that regulate it are studied as possible targets for the treatment/prevention of loss of muscle mass in diverse muscle atrophying conditions [1,2]. However, the exact mechanism by which S6K1 regulates muscle mass and metabolism remains to be identified. Substrates of S6K1 proposed to mediate its actions are all factors that associate with or regulate mRNA translation initiation. These include the ribosomal protein S6 (S6) and the eukaryotic mRNA translation initiation factor 4B (eIF4B), both of which upon activation induce mRNA translation initiation. S6K1 also phosphorylates eukaryotic mRNA translation elongation factor 2 (eEF2) kinase, an inhibitor of mRNA translation (reviewed in [3,4]). In skeletal muscle, concurrent increase in phosphorylation of S6K1, S6 and eIF4B are observed in conditions that stimulate muscle protein synthesis, including resistance exercise, provision of amino acid, and stimulation with insulin/IGF-1 [1,5,6]. However, the functions/regulation of these substrates do not account for the actions of S6K1 in controlling mRNA translation initiation and muscle mass [6,7], suggesting a role for other substrates of this kinase.

Programmed cell death 4 (PDCD4), (also known as MA3, TIS (topoisomerase inhibitor-suppressed) [8], H731 [9], and interleukin-12 inducible human gene 197/15a [10] (reviewed in [11])), is a more recently discovered substrate of S6K1 [12]. In the hypo phosphorylated state, it binds to both eIF4A and eIF4G, leading to both the inhibition of the helicase activity of eIF4A and of the formation of eIF4F complex. These changes will lead to the suppression of translation of mRNA with secondary structures at their 5′-UTR ends [13,14]. Upon mitogen stimulation, activated S6K1 phosphorylates Ser67 in PDCD4. This targets it for ubiquitination by the ubiquitin protein ligase beta-transducin repeat containing protein (β-TRCP) and subsequent degradation by the proteasome [12].

Much of what is known about PDCD4 is from cancer studies where PDCD4 is proposed to function as a cell cycle inhibitor/tumor suppressor. Loss of this protein is associated with invasion, progression or increased aggression of numerous, but not all [15], cancers, including ovarian [16], lung [17], breast [18], liver [19] and colon cancers [11]. As a substrate of mTORC1/S6K1, PDCD4 may mediate the effect of this kinase pathway on protein synthesis in skeletal muscle. However, not much is known about the role or regulation of PDCD4 in muscle, the tissue that is quantitatively the most important in whole body protein metabolism. It was recently shown that the abundance of PDCD4 in rat skeletal muscle is sensitive to feeding and food deprivation cycle: its abundance increased in skeletal muscle of food-deprived rats, but in fed or refed rats, its abundance decreased along with increase in muscle fractional protein synthesis [20]. These data suggest that interventions that regulate PDCD4 abundance may be explored in the treatment of muscle wasting, a feature of diseases like cancer, AIDS, and trauma. However this study was mainly correlative and did not examine whether or not mTORC1/S6K1 is required for PDCD4 regulation in muscle.

In the present work, using L6 myotubes, our specific objectives were to: 1) examine the requirement for mTORC1/S6K1 and the ubiquitin proteolytic system in regulating PDCD4; 2) examine the contribution of amino acids vs. growth factors in mediating the effect of nutrition on PDCD4; and 3) determine whether nutritional status affects the interaction of PDCD4 with components of eIF4F. Because others have suggested that signalling pathways that regulate protein metabolism may be regulated differently in myotubes versus myoblasts [21] and because the regulation of PDCD4 may depend on cell type [22], we also assessed the effect of PDCD4 depletion by RNA interference (RNAi) on myotube total and myofibrillar protein synthesis.

## Results

### Abundance of PDCD4 in L6 myotubes is sensitive to medium composition and requires mTORC1 and the proteasome

**Figure 1:**
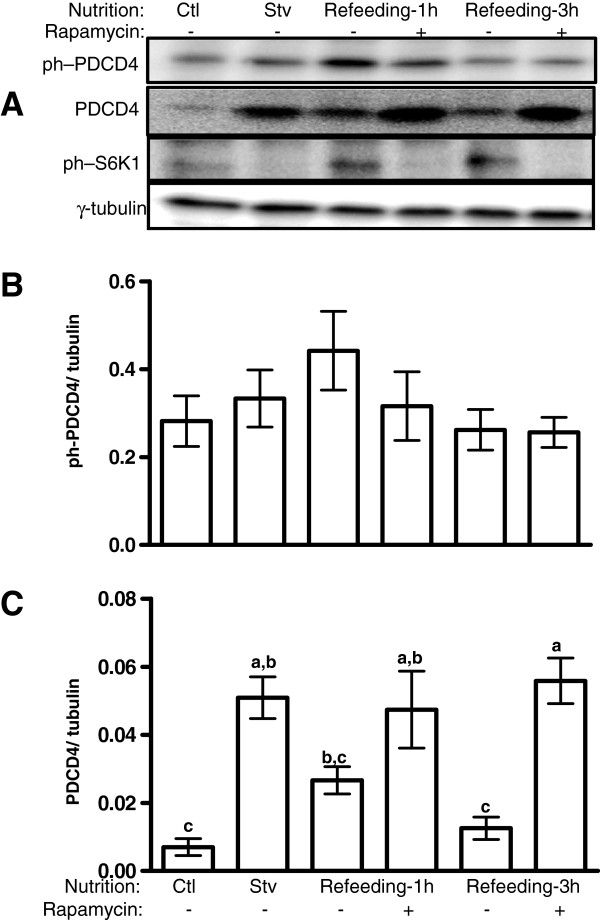
**In L6 myotubes, PDCD4 abundance, but not phosphorylation, is sensitive to medium nutrient composition and requires mTORC1 function.** L6 Myotubes were incubated in differentiation (Ctl) or starvation medium (serum-free, amino acid-free RPMI medium) for 12 h. They were then harvested (Stv) or refed in the differentiation medium supplemented with DMSO (−) or 50 nM rapamycin (+) for 1 or 3 h. **(A)** Phosphorylated (ph) and total PDCD4 (Ser67), ph-S6K1 (Thr389) and γ-tubulin were analyzed by immunoblotting. **(B** and **C)** phosphorylated and total PDCD4 signals were expressed relative to γ tubulin. Means ± SE of 5–6 independent experiments; bars with different letters differ (*P* < 0.05).

Given the identification of PDCD4 as a substrate of mTORC1/S6K1 signalling, and the fact this kinase pathway is regulated by nutrients, we examined the effect of nutrient deprivation on the regulation PDCD4 in L6 myotubes. Neither 12 h of serum and amino acid deprivation nor refeeding in a complete medium had any significant effect on PDCD4 Ser67 (the residue targeted by S6K1) phosphorylation (Figure 1A, B). Furthermore, serum and amino acid deprivation had no effect on phosphorylation on Ser457, although phosphorylation on this residue was increased by refeeding (see Additional file 1). However, PDCD4 abundance increased more than four-fold in starved cells and decreased progressively with time during refeeding such that by 3 h of refeeding, values in re-fed cells were not different from control (P < 0.05, Figure 1A, C). Incubation with rapamycin, an mTORC1 inhibitor, abolished the effect of re-feeding on PDCD4 abundance (Figure 1A, C).

**Figure 2:**
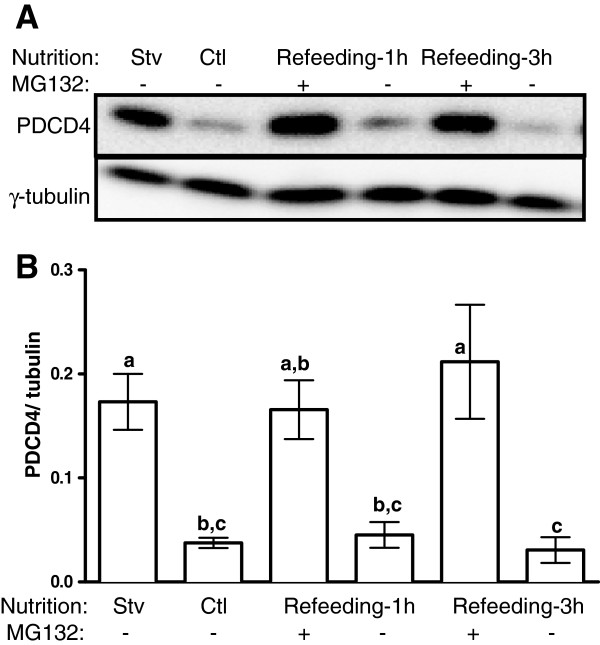
**Abundance of PDCD4 in myotubes is regulated by the proteasome.** L6 myotubes were incubated in differentiation (Ctl) or starvation medium as described in Figure 1. They were then harvested (Stv) or refed in the differentiation medium supplemented with DMSO (−) or 10 μM MG132 (+) for 1 or 3 h. **(A)** Total PDCD4 and γ tubulin were analyzed by immunoblotting. **(B)** PDCD4 signals were quantified and expressed relative to γ-tubulin. Means ± SE; n = 4–6; bars with different letters differ (*P* < 0.05).

Because the ubiquitin system is implicated in the phosphorylation-dependent degradation of PDCD4, we incubated the cells with MG132, a proteasome inhibitor. Like rapamycin, addition of MG132 prevented the disappearance of PDCD4 during re-feeding (Figure 2A, B).

### Growth factors, but not amino acids, regulate PDCD4 abundance

**Figure 3:**
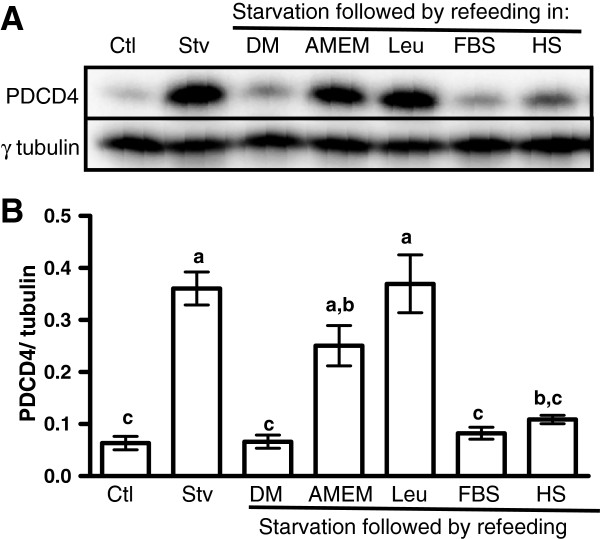
**Growth factors, but not amino acids, promote the degradation of PDCD4.** L6 myotubes were incubated in differentiation (Ctl) or starvation medium as described in Figure 1. They were then harvested (Stv) or refed in the differentiation medium (DM), serum-free AMEM (AMEM), or in starvation medium supplemented with either 0.4 mM leucine (LEU), 10% dialyzed FBS (FBS) or 2% horse serum (HS) for 1 h. **(A)** Cells were harvested and analyzed by immunoblotting. **(B)** PDCD4 signals were quantified and expressed relative to γ-tubulin. Means ± SE; n = 4; bars with different letters differ (*P* < 0.05).

The experiments above did not indicate whether the observed effects of refeeding were due to nutrients or growth factors. To address this question, we repeated the starvation experiment but re-fed the myotubes in either the differentiation medium, serum-free AMEM, or starvation medium supplemented with leucine, dialyzed FBS or horse serum. Only media that contained serum promoted the degradation of PDCD4 (Figure 3A, B).

### Association of PDCD4 with eIF4A in L6 myotubes is sensitive to medium composition

**Figure 4:**
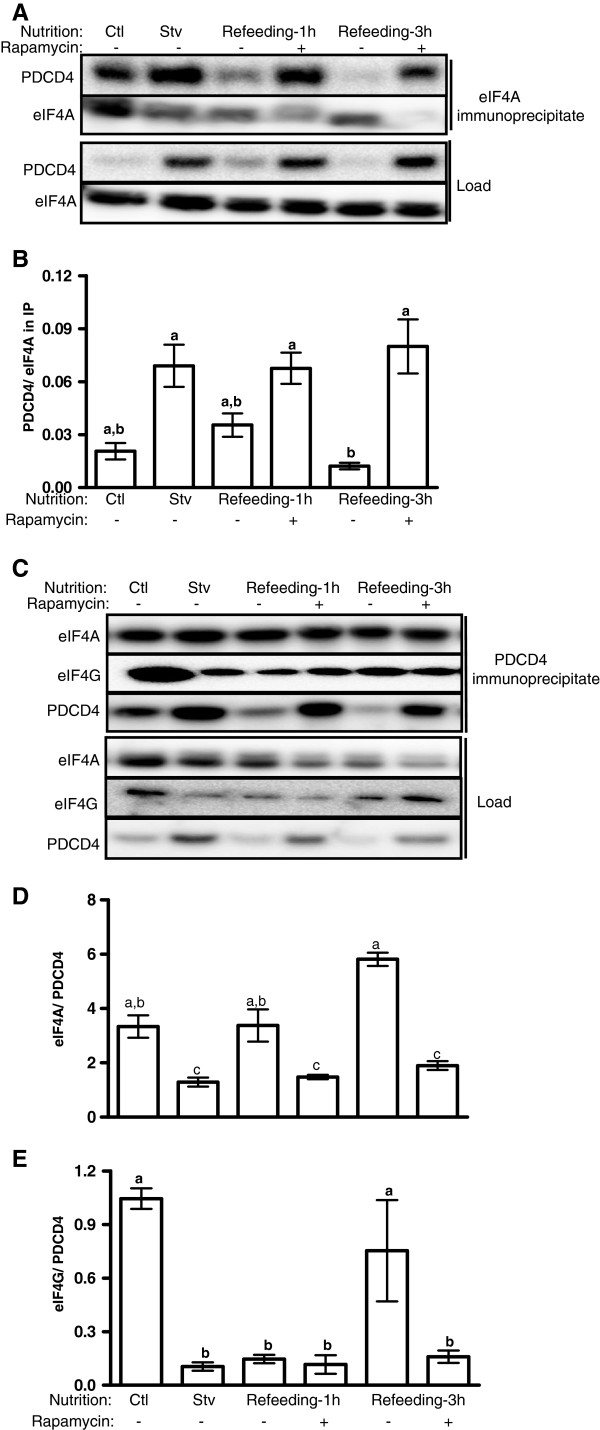
**Interaction of PDCD4 with eIF4A or eIF4G in myotubes.** Myotubes were incubated in differentiation (Ctl) or starvation (serum- and amino acid-free) medium for 12 h. They were then harvested (Stv) or refed in the differentiation medium in the presence of rapamycin for 1 or 3 h. Lysates were immunoprecipitated with either **(A)** anti eIF4A or **(C)** anti PDCD4 antibodies followed by western blotting. Signals from immunoprecipitates were quantified and shown in **B**, **D** and **E**. Means ± SE; n = 4; bars with different letters differ (*P* < 0.05).

PDCD4 inhibits mRNA translation initiation at least in part by its binding to eIF4A and eIF4G. The amount of PDCD4 found in eIF4A immunoprecipitate was increased by starvation but fell gradually during refeeding, especially at 3 h, at which time the values were not different from those observed in fed cells (Figure 4A, B). In another experiment, we carried out the reciprocal immunoprecipitation. The amount of eIF4A in PDCD4 immunoprecipitate was unchanged by treatments; however, because starvation increased PDCD4 abundance in the immunocomplex, the ratio of eIF4A to PDCD4 was suppressed by starvation. This was reversed by refeeding (Figure 4C-D). In addition, the pattern of eIF4G association with PDCD4 was similar to that observed for eIF4A; however, the effect of refeeding was not seen until the 3 h time point (Figure 4C and 4E). Finally we examined the effects of mTORC1 inhibition on the interactions. In all cases, the effect of refeeding on the interactions of PDCD4 with eIF4A and 4G was sensitive to rapamycin (Figure 4A-E).

### PDCD4 depletion in myotubes had modest effect on protein synthesis

**Figure 5:**
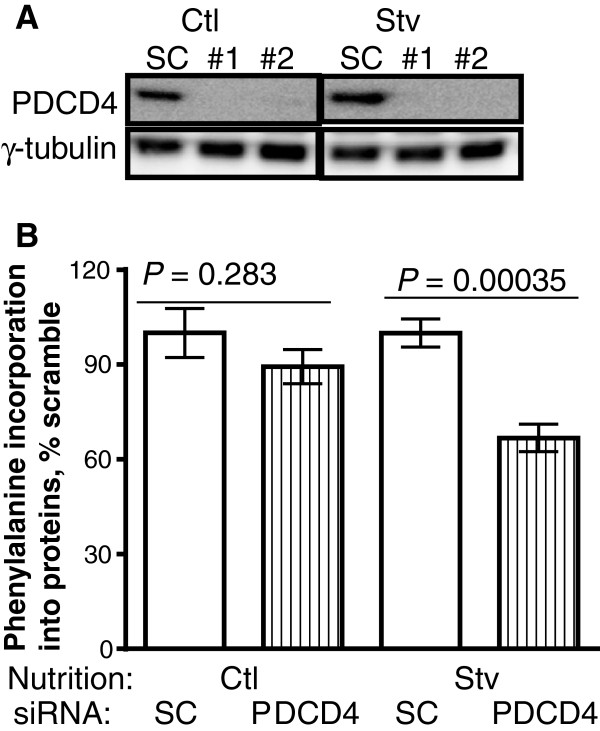
**Phenylalanine incorporation into proteins in myotubes depleted of PDCD4. (A)** PDCD4 abundance in cells treated with scrambled siRNA or siRNA oligonucleotides designed against PDCD4. **(B)** Cells treated with scrambled (SC) or PDCD4 siRNA #2 were cultured in differentiation (Ctl) or serum-free medium (Stv) for 12 h. Phenylalanine incorporation into proteins was then measured. Within each nutrition group, cells depleted of PDCD4 were compared to those treated with scrambled siRNA using paired *t*-test. Means ± SE of 5–6 independent experiments.

To examine the significance of PDCD4 in regulating myotube mixed protein synthesis, we used RNAi to deplete this protein (Figure 5A) and then measured incorporation of phenylalanine into myotube proteins. In fed cells (Ctl), incorporation of phenylalanine into mixed proteins in cells deprived of PDCD4 was not different from the value in those treated with scrambled oligonucleotides (Scrambled siRNA: 2148 ± 165; PDCD4 siRNA #1: 1924 ± 91, PDCD4 siRNA #2: 1919 ± 115 cps/mg protein, P > 0.5, Figure 5B). In cells deprived of serum but supplied with amino acids (i.e., serum-free AMEM), phenylalanine incorporation into proteins in cells treated with PDCD4 siRNA #1 was 86% of values in those treated with scrambled siRNA (P = 0.11, data not shown); the values in those treated with PDCD4 siRNA #2 was 67% of those treated with scrambled siRNA (P = 0.00035, Figure 5B). In another experiment, PDCD4 deprived cells were incubated in medium lacking both serum and amino acids (RPMI 1640). Incorporation of phenylalanine into myotube total mixed proteins in cells treated with the two PDCD4 siRNA oligonucleotides was 72-80% of the values in cells treated with scrambled siRNA oligonucleotides (data not shown). Finally we examined the effect of PDCD4 on the regulation of myofibrillar proteins. Depletion of PDCD4 led to a 30% reduction in phenylalanine incorporation into myofibrillar proteins (see Additional file 2).

**Figure 6:**
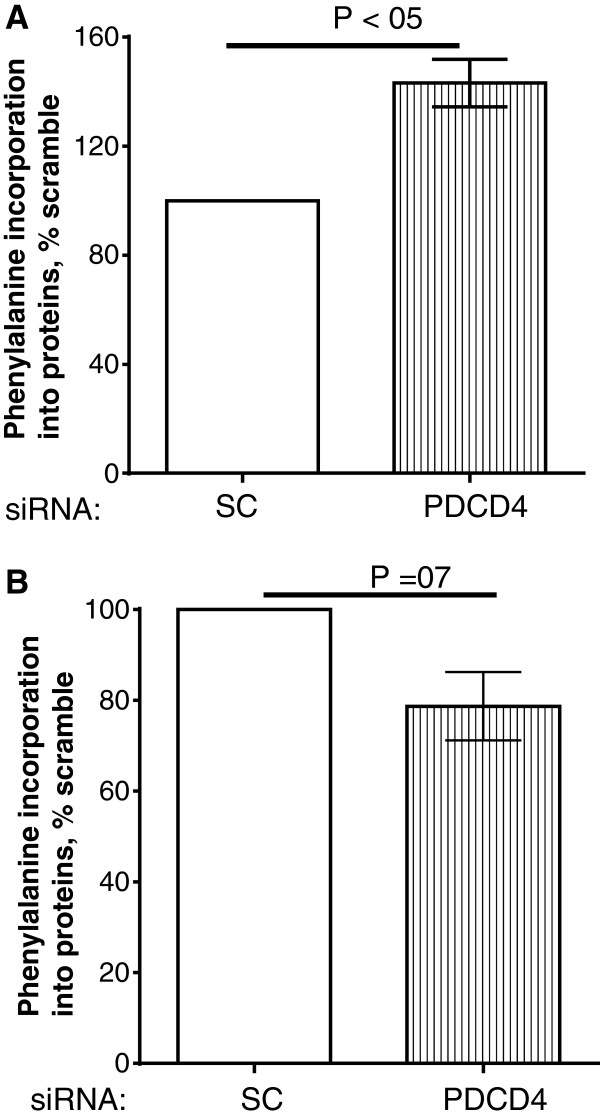
**Phenylalanine incorporation into proteins in myoblasts and myotubes depleted of PDCD4. (A)** L6 myoblasts were treated with scrambled (SC) or PDCD4 oligonucleotides as described in Figure 5. Cells were then deprived of amino acids and serum for 12 h, after which phenylalanine incorporation into proteins was measured. **(B)** Myotubes were treated as in Figure 5 except that siRNA oligonucleotides obtained from Life Technologies were used. Data are expressed as % of SC. Means ± SE of 3–4 independent experiments.

The finding of reduced protein synthesis in cells deprived of PDCD4 was surprising given the inhibitory role of this protein on mRNA translation and our previous finding in myoblast. Thus we carried out two additional control experiments. First, we repeated the myoblast experiments and showed that as before, in starved cells, PDCD4 depletion increased protein synthesis by 43% (Figure 6A). Finally, we used siRNA oligonucleotides purchased from another company (Life Technologies, Burlington Ontario Canada) to silence PDCD4 in myotubes. Protein synthesis in myotubes deprived of PDCD4 was reduced by 21% (P = 0.07, Figure 6B).

**Figure 7:**
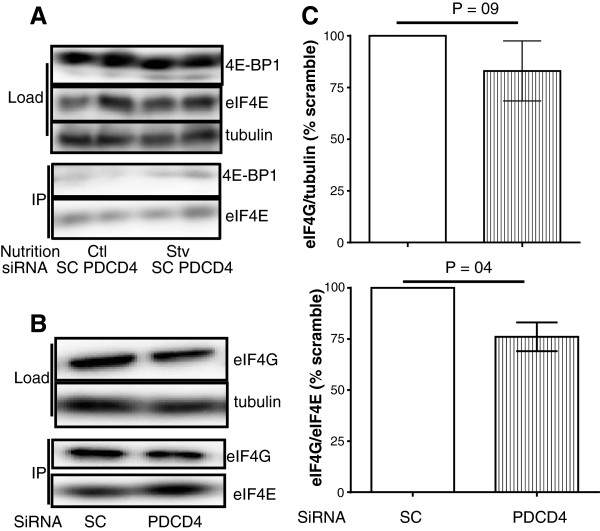
**Abundance of translation initiation factors and interaction of eIF4G with eIF4E in myotubes depleted of PDCD4.** Cells were treated with scrambled (SC) or PDCD4 oligonucleotides as in Figures 5 and 6. They were then deprived of amino acids and serum for 12 h. **(A)** Abundance of 4E-BP1 and eIF4E, and interaction of the two were measured. Reduced phosphorylation of 4E-BP1 is obvious in downward shifts of signals in lanes for starved cells (compare lanes 1 and 2 to lanes 3 and 4 in the top panel of Figure 7A). **(B)** Abundance of eIF4G and its interaction with eIF4E. **(C)** Quantification of eIF4G abundance (upper chart; tubulin served as loading control) and its interaction with eIF4E (lower). Data are expressed as % of SC. Means ± SE of 3 independent experiments.

To gain insight into the mechanisms of effect of PDCD4 knockdown on myotube protein synthesis, we examined the regulation of components of mTORC1 signalling and mRNA translation initiation. Although starvation predictably reduced the phosphorylation of 4E-BP1 and increased the binding of 4E-BP1 to eIF4E, PDCD4 depletion had no effects on these parameters (Figure 7A). Likewise, in starved myotubes, PDCD4 depletion had no effect on S6K1 or S6 phosphorylation (data not shown). However, there was a trend towards reduced eIF4G in cells depleted of PDCD4 (17% lower compared to Scramble, P = 0.09). Furthermore, PDCD4 depletion significantly reduced eIF4G interaction with eIF4E (Figure 7B and C).

## Discussion

In this study, we demonstrated that in myotubes, the regulation of PDCD4 abundance was reversibly modified by a starvation-refeeding cycle. Collectively, the data presented here are the first evidence to demonstrate a requirement for mTORC1 and the proteasome in regulating the abundance of PDCD4 in muscle cells. We also presented evidence that, at least in myotubes, in the absence of growth factors, amino acids had little effect in regulating the abundance of this protein. Finally, in starved myotubes, and contrary to observations in myoblasts [20] and non muscle cells [12], depletion of PDCD4 had minimal effect on the incorporation of phenylalanine into myotube proteins. Rather, in starved myotubes, PDCD4 depletion further reduced eIF4G binding to eIF4E.

In spite of the fact that PDCD4 has been characterized as a substrate of S6K1 and an inhibitor of cap-dependent mRNA translation initiation, there is a paucity of information on the significance of PDCD4 in skeletal muscle. Also, it is unknown if the regulation of PDCD4, like mTORC1/S6K1, is sensitive to nutrients. In the present study, Ser67 and Ser457 phosphorylation of PDCD4 correlates poorly with its abundance. A requirement for mTORC1/S6K1 in regulating PDCD4 abundance suggests that PDCD4 may be phosphorylated on additional residues. However, PDCD4 degradation appears to depend specifically on Ser67 phosphorylation [12]. It is also possible that phosphorylated PDCD4 does not accumulate because degradation by the proteasome is very rapid. However, in refed cells treated with MG132, Ser67 phosphorylated PDCD4 did not accumulate to a greater extent in comparison with cells not treated with the drug (data not shown).

Although amino acids can activate mTORC1 [23,24], the effects of amino acids require some amount of insulin [25-28]. Our finding that leucine or a medium that contained all the 20 amino acids but lacked growth factors had insignificant effects on PDCD4 abundance is consistent with this view. AKT too may phosphorylate PDCD4 and target it for degradation [29]. In fact, a requirement for serum rather than amino acids might implicate AKT rather than mTORC1/S6K1 in the phosphorylation and degradation of PDCD4 since AKT does not require amino acid for its activation [30]. However, incubation with rapamycin would not only inhibit mTORC1/S6K1 but should lead to a greater activation of PI3K-AKT pathway due to the loss of negative inhibition conveyed by activated S6K1 (reviewed in [31]). In our study, the fact that inhibition with rapamycin during a 1-h refeeding completely prevented the disappearance of PDCD4 clearly suggests that mTORC1/S6K1 is the main pathway that targets PDCD4 for degradation in myotubes.

Our data showing that PDCD4 knock down suppressed incorporation of phenylalanine into myotube mixed proteins are surprising, given the characterization of the protein as an mRNA translation initiation inhibitor. Furthermore, depletion of PDCD4 in myoblasts ([20] and Figure 6) and in non muscle cells [12] increases protein synthesis. A possible explanation might be that the regulation of myofibrillar proteins, the predominant proteins in myotubes, is different from that of total protein. However, we showed that incorporation of phenylalanine into myofibrillar proteins in cells depleted of PDCD4 was 30% lower compared with cells with normal level of PDCD4 (see Additional file 2). We did not measure the rate of synthesis of sarcoplasmic proteins; nevertheless, our data showing a suppression of phenylalanine incorporation into total and myofibrillar proteins suggest that even if depletion of PDCD4 increased the synthesis of sarcoplasmic proteins, such an increase was likely too small to offset the decrease in myofibrillar protein synthesis. It is not clear how PDCD4 depletion would regulate eIF4G abundance and interaction with eIF4E, although there is evidence that PDCD4 can transcriptionally regulate the abundance of some proteins [11]. However, there is no evidence that eIF4G is one of such proteins.

Combined with data from myoblasts and non muscle cells, our data suggest that the effect of PDCD4 on protein synthesis may depend on cell type and/or stage of development, as previously suggested [22]. In this regard, although PDCD4 has been implicated in regulating the abundance of some proteins, including p21 (Waf1/Cip1) [32] and lysyl oxidase [33], only c-myb [34], procaspase-3 [35] and p53 [36] have been demonstrated as natural mRNA translation substrates of PDCD4. These are all factors involved in regulating cell proliferation and migration, and therefore of more relevance in proliferating cells. This is consistent with the notion that the effect of PDCD4 on mRNA translation and protein synthesis might depend on the physiological state of the cell. However, PDCD4 and its targets may still be relevant in regulating muscle protein synthesis and mass during muscle development and regeneration. For example during muscle hypertrophy or repair following injury, satellite cells need to be activated, leading to the proliferation of myoblasts that will subsequently fuse to form myotubes [37]. These can then fuse with existing myofibers or be used to form new fibers [38,39]. PDCD4 might be involved in this regulation. Consistent with this, abundance of PDCD4 increases during initiation of L6 differentiation into myotubes (Abdullahi A. and Adegoke O.A.J., unpublished observations).

## Conclusions

We showed that in L6 myotubes, the regulation of PDCD4 abundance by nutritional factors is sensitive to mTORC1 and ubiquitin dependent proteolytic system. In the absence of growth factors, amino acids, including leucine, appear to play a minor role in regulating PDCD4 abundance. Unlike in proliferating myoblasts and non muscle cells, depletion of PDCD4 had, at best, only modest effect on myotube protein synthesis, indicating that the effect of PDCD4 in muscle cells is dependent on the physiological state of the cell. Additional studies are needed to dissect the mechanisms behind these differential effects of PDCD4.

## Methods

### Reagents

Fetal Bovine Serum (FBS), Horse Serum (HS), Lipofectamine RNAi^Max^, Opti-MEM medium, and antibiotic/antimycotic reagents were purchased from Life Technologies (Burlington, Ontario Canada). Amino acid-free medium (RPMI 1640) was obtained from US Biological (Swampscott MA, USA). PDCD4 siRNA oligonucleotides, phosphatase and protease inhibitor cocktails were purchased from Sigma-Aldrich (St. Louis, MO, USA). Alpha Modification of Eagle’s Medium (AMEM) was obtained from Wisent (St-Bruno, Quebec, Canada).

### Antibodies

Antibodies to eIF4A, eIF4G, phosphorylated (Thr389) S6K1, and horseradish peroxidase (HRP)-conjugated secondary antibodies (anti-rabbit and anti-mouse) were purchased from Cell Signaling Technology (Danvers, Massachusetts, USA). Antibody against PDCD4 was from Cell Signaling Technology or Santa Cruz Biotechnology (Santa Cruz, California, USA). Antibodies against phosphorylated (Ser67, Ser457) PDCD4 were from Sigma-Aldrich or Aviva Systems Biology (San Diego, California, USA).

### Cell culture

L6 myoblasts were cultured in 12-well plates in growth medium (GM: AMEM supplemented with FBS and antibiotic-antimycotic agents to final concentrations of 10 and 1%, respectively) until they reached ~80% confluency. Cells were then shifted into differentiation medium (DM: AMEM as above except that 2% HS replaced 10% FBS). Experiments were carried out on day 4–5 of differentiation. For starvation experiments, myotubes were grown in differentiation medium or starved in amino-acid free, serum-free medium (amino acid-free RPMI 1640) for 12 h. They were then refed in DM for 1 or 3 h. To examine the roles of amino acids and growth factors in regulating PDCD4 abundance, in some experiments refeeding was done in incubation media of varied composition. To examine the requirement for mTORC1 or the ubiquitin dependent proteolytic system on the regulation of PDCD4, additional refeeding experiments were carried out in the presence of inhibitors of these pathways (50 nM rapamycin and 10 μM MG132, respectively) or equivalent volumes of DMSO. At the end of the experiments, cells were harvested in a lysis buffer (25 mM Tris, pH 7.5, 1 mM EDTA, 2% (w/v) sodium dodecyl sulphate (SDS), 1 mM DTT, supplemented with protease (10 μl/mL) and phosphatase (10 μl/mL) inhibitor cocktails).

### RNA interference

Myotubes on day 4 of differentiation were transfected with 30 nM siRNA oligonucleotides designed against PDCD4 or with a proprietary scrambled oligonucleotide ((Sigma Aldrich, Oakville, Ontario Canada)), using Lipofectamine RNAi^Max^ (Life Technologies) as previously described [20]. We used the following PDCD4 siRNA oligonucleotides: PDCD4 #1 sense (GUCUUCUACUAUUACCAUA [dT] [dT]), PDCD4 #1 antisense (5′UAUGGUAAUAGUAGAAGAC [dT] [dT]), PDCD4 #2 sense (CUACUAUUACCAUAGACCA [dT] [dT]), and PDCD4 #2 antisense (UGGUCUAUGGUAAUAGUAG [dT] [dT]. Thirty eight hours after transfection, cells were cultured in DM or starvation medium. Phenylalanine incorporation into proteins was then measured by assessing the incorporation of radioactive phenylalanine into trichloroacetic acid (TCA) precipitable proteins [29,40].

### Western blotting and immunoprecipitation

Proteins were resolved on 7.5, 10, or 15% SDS-PAGE (depending on the antigens being studied), transferred onto polyvinylidene difluoride (PVDF) membranes, which were then immunoblotted for the indicated antigens, as previously described [20]. Immunoblot signals were quantified using the Carestream Molecular Imaging software (version 5.0.2.30, Carestream Health Inc., Rochester, New York, USA).

To immunoprecipitate eIF4A or PDCD4, myotubes were cultured in 10-cm plates. Following appropriate treatments, cells were rinsed in ice-cold PBS and then lysed in 500 μl of ice-cold lysis buffer (40 mm HEPES (pH 7.5), 120 mM NaCl, 1 mM EDTA, 10 mM pyrophosphate, 10 mM glycerol 2 phosphate, 0.5 mM orthovanadate) supplemented with 0.03% CHAPS, 1 mM DTT, 0.5 mM NaV, 1 mM benzamidine, 6.25 mM N-ethyl-maleimide (NEM) and protease inhibitor cocktail (10 μL/mL). One hundred micrograms of myotube proteins were combined with either anti eIF4A or anti-PDCD4 (Santa Cruz biotechnology) antibodies and the mix rotated overnight at 4°C. The following day and in order to precipitate the antigen-antibody complex, 50 μL of re-suspended BioMag protein G–bound beads (GE Healthcare, Baie d’Urfe, Quebec, Canada) were added to each of the immunoprecipitation tubes and the suspension rocked gently at 4°C for 1 h. The beads were collected on a magnetic stand (Life Technologies) and washed 3 times with 0.1 M sodium phosphate buffer. After the last wash, beads were re-suspended in 1X SDS-PAGE sample buffer and boiled at 95°C for 2 minutes. Following a brief centrifugation, eluates were collected, separated on 10% SDS-PAGE, and blotted for PDCD4 and eIF4A.

### Statistics

Data are presented as means ± SEM. Treatment means were compared using a one-way analysis of variance (ANOVA) and differences among individual means assessed using the Bonferroni multiple comparison test or, as in Figures 5, 6 and 7, by paired Students T-tests. Analyses were done using GraphPAD (Version 6, GraphPAD Software, La Jolla California, USA). The level of significance was set at P < 0.05.

## Competing interest

The authors declare that they have no competing interest.

## Authors’ contributions

OAJA conceived, designed, conducted experiments, wrote and reviewed the manuscript; DK, NI and NM conducted experiments and reviewed the manuscript. All authors read and approved the final version of the manuscript.

## Supplementary Material

Additional file 1 PDCD4 phosphorylation (Ser457) in L6 myotubes. Click here for file

Additional file 2 Myofibrillar protein synthesis in L6 myotubes. Click here for file
