# Supplementary material for: Differential effects of PDCD4 depletion on protein synthesis in myoblast and myotubes
Source: BMC Cell Biol. 2014 Jan 9;15:2. doi: 10.1186/1471-2121-15-2 (PMC3893489; doi:10.1186/1471-2121-15-2)
Supplement: Additional file 1 — PDCD4 phosphorylation (Ser457) in L6 myotubes. [file 1471-2121-15-2-S1.pdf]

Additional file 1

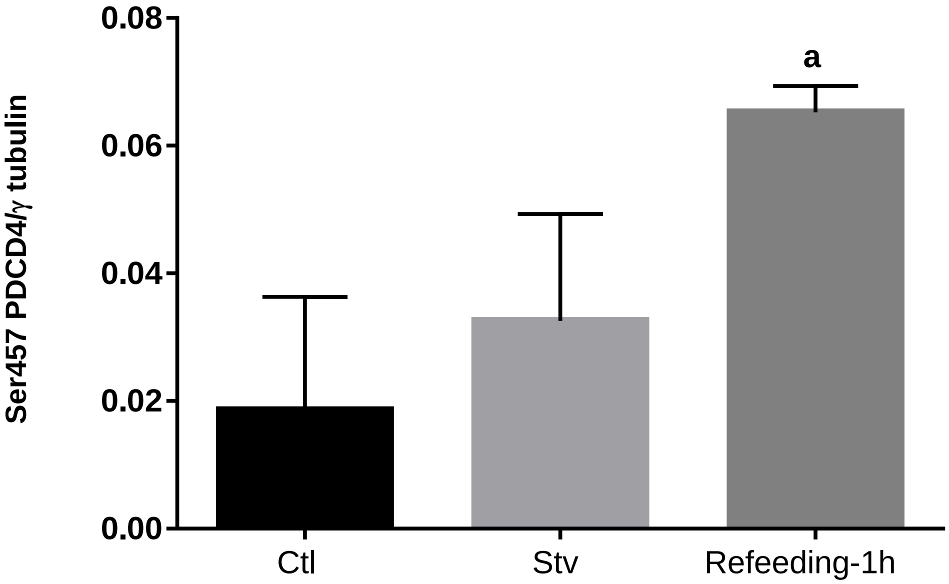

Additional file 1: L6 Myotubes were incubated in differentiation (Ctl) or starvation medium (serum-free, amino acid-free) for 12 h. They were then harvested (Stv) or refed in the differentiation medium for 1 h. Phosphorylated (ph, Ser457) PDCD4 and γ-tubulin were analyzed by immunoblotting. Mean ± SE, n = 3, [a]P <0.05 versus Ctl.
