# Supplementary material for: Differential effects of PDCD4 depletion on protein synthesis in myoblast and myotubes
Source: BMC Cell Biol. 2014 Jan 9;15:2. doi: 10.1186/1471-2121-15-2 (PMC3893489; doi:10.1186/1471-2121-15-2)
Supplement: Additional file 2 — Myofibrillar protein synthesis in L6 myotubes. [file 1471-2121-15-2-S2.pdf]

Additional file 2

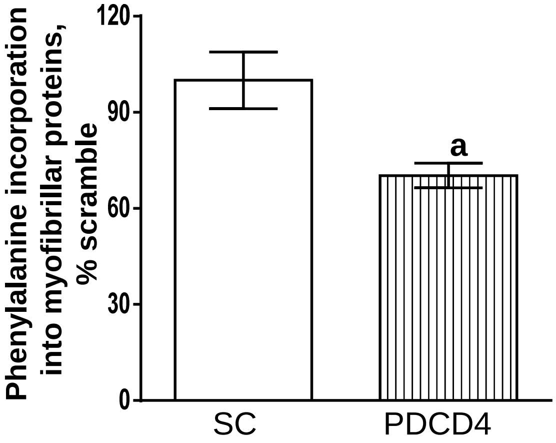

Additional file 2: Three days after the change of medium from growth to differentiation medium, myotubes were transfected with scramble (SC) or PDCD4 siRNA oligonucleotides. Two days after transfection (day 5 of differentiation), phenylalanine incorporation into myofibrillar proteins was measured as described by Naggar et al 2004 (Kobe J Med Sci 50 (2): 39-46). Mean ± SE, n = 3, [a]$P<0.05$ compared to SC.
